# Annotations

**Published:** 1907-11-09

**Authors:** 


### ANNOTATIONS.

### The Infants' Hospital.

In the new buildings of the Infants' Hospital in Vincent Square, which have just been opened for the reception of patients, there are one or two unusual features. Especially noteworthy is the absence of an operating theatre. The reason given for this apparent omission is that there will be no necessity for such a provision, because the beds are intended only for infants suffering from disorders of nutrition and of the gastro-enteric system. But there are a certain number of infantile disorders of this kind, for which operative treatment may be necessary when other methods have failed. It is only necessary to mention stenosis of the pylorus and pyloroplasty as an instance. It is further to be noted that there is no out-patients' department, on the ground that the best methods of treatment for these conditions can only be afforded to in-patients. This attitude is quite a reasonable one, but it must necessitate admission of patients very largely by recommendation from medical practitioners in the immediate district. What, then, do the authorities of the Infants' Hospital propose to do with a baby, sent in possibly by a doctor late at night under a diagnosis of enteritis, which is there diagnosed as suffering from intussusception? By the time the resident at Vincent Square has made up his mind to ask the advice of his consultant from Cavendish Squareland, and their united skill has clinched such a diagnosis, do they propose to send on the child to the Westminster or some other General Hospital, where the same process, involving a delay of some hours, and further exposure for the baby, must again be gone through before an operation can be done? We trust not, and we think a small room fitted with the very simple necessaries for an occasional operation ought certainly to be provided.

### Dangerous Prescriptions.

The rule that when prescribing arsenic and strychnine together and in solution, an acid, not an alkaline solution of the arsenic, should be selected, has its due place in the current manuals of materia medica and pharmacy. Nevertheless there is reason to believe that it is not infrequently neglected, and sometimes at least with apparent impunity. The danger is that in the presence of an alkali the strychnine salt is decomposed and the alkaloid precipitated. Hence the later doses of the mixture are apt to contain the greater part, or even the whole, of the strychnine, with obvious risks to the unfortunate patient. Doubtless when the doses are very small, and when, as is a not infrequent habit, the medicine bottle is well shaken before the dose is poured out, the risk is reduced to a minimum, and it is to these circumstances that the absence of evil consequences, when the above combination is practised, must be attributed. Every now and then, however, a case is reported which shows that danger exists, and thus enforces the lesson that the prescription of arsenic and strychnine in solution should be framed in accordance with sound chemical principles. A recent instance, and one unfortunately attended with a fatal issue, is that of a woman to whom was given a prescription for equal parts of Liq. arsenicalis and Liq. strychninæ, with the direction to take six drops in water thrice daily. The dispenser supplied an ounce of the mixture, and one bottle of this was taken without evil result. When the second bottle was almost empty the patient, in order as it were to take the last dose, filled the bottle with water and then drank the contents. Soon after she had symptoms of strychnine poisoning, and in the course of two hours or so she died. This melancholy result was thus due to the breach of a well-established and oft-proclaimed rule. Solution of strychnine may quite appropriately be combined with the hydrochloric solution of arsenic, but to order the alkaline (Fowler's) solution with strychnine is to present the patient with an essentially dangerous prescription. Neither prescriber nor dispenser should sanction such a combination.

### Diet and Personal Idiosyncrasy.

The dietetic habits of mankind are favourite subjects of comment and criticism, and they appear to exercise as great a fascination for the social reformer and the faddist as they do for the physiologist and the physician. But neither the amateur prescriber nor the scientific worker appears to produce much practical result. Custom and habit continue their sway, and though gradual modifications are introduced, these seem to be the result of a blind instinctive judgment rather than of scientific argument and analysis. In this respect the contrast afforded by the food habits of different nations is a subject of considerable interest. Differences both in form and substance are obvious, yet for these it is difficult to suggest any adequate explanation. The probability would appear to be that the accumulated experience of successive generations produces an intuitive appreciation of those dietetic modifications which are best adapted to climatic and other influences in the national environment, and that it is this experience rather than a process of reasoning which determines food customs and practices. In a recent paper Dr. Chas. J. Macalister has transferred the enquiry from nations to individuals, and has endeavoured to account for the food peculiarities of persons who do not flourish on the diet customary to their fellows. He proposes as an explanation a reversion to some ancestral type with a corresponding limitation of metabolic capacity, and thus finds in the purely vegetable feeder an example of a well-known biological manifestation. This is certainly an interesting suggestion, though it hardly advances the diet question in any practical respect. But it does tend to emphasise the fact that foods and feeding depend on many issues, and that some of these, at all events, cannot be weighed and measured in the laboratory. Further, man's extension and survival on the surface of the earth have been largely due to his capacity to adapt himself to varying climatic and other influences in his environment. The prescribers of certain rigid diet schemes appear to forget that this ability extends also to the nature and quality of the food supply.

